# Supplementary material for: Haplotype Analysis of GmSGF14 Gene Family Reveals Its Roles in Photoperiodic Flowering and Regional Adaptation of Soybean
Source: Int J Mol Sci. 2023 May 29;24(11):9436. doi: 10.3390/ijms24119436 (PMC10253954; doi:10.3390/ijms24119436)
Supplement: Supplementary file 1 [file ijms-24-09436-s001.zip › ijms-2384071-supplementary.pdf]

Supplementary Materials

This PDF file includes:

Supplementary Figures S1-S2

Supplementary Tables S1-S4

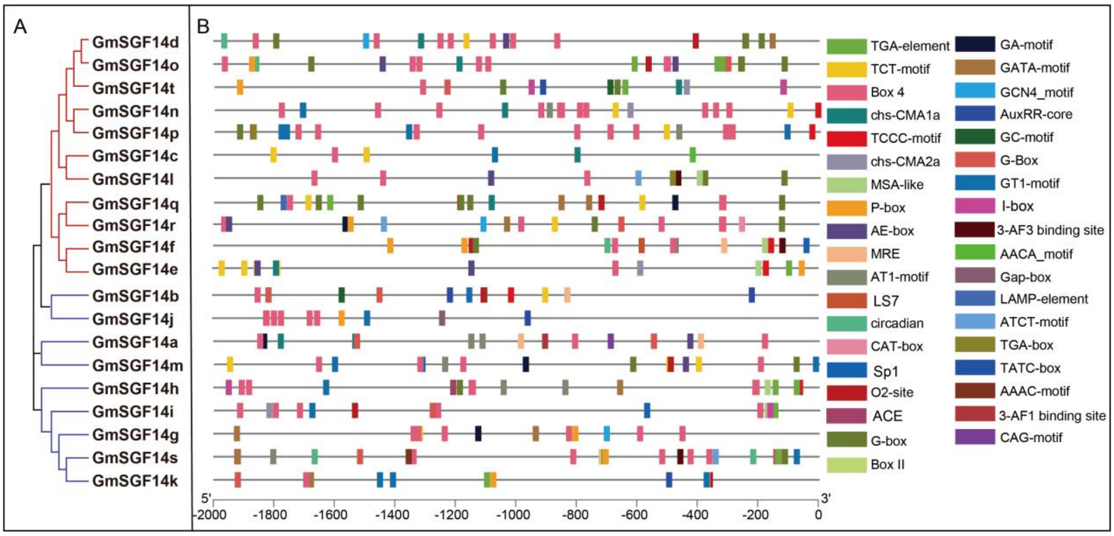

Figure S1. Analysis of cis-acting elements within the promoters of *GmSGF14* genes.(A) Phylogenetic tree of soybean GmSGF14 proteins, with different subfamilies represented by different colors. (B) Type and location of cis-acting elements in the promoter region. Different cis-elements are represented by different colored boxes.

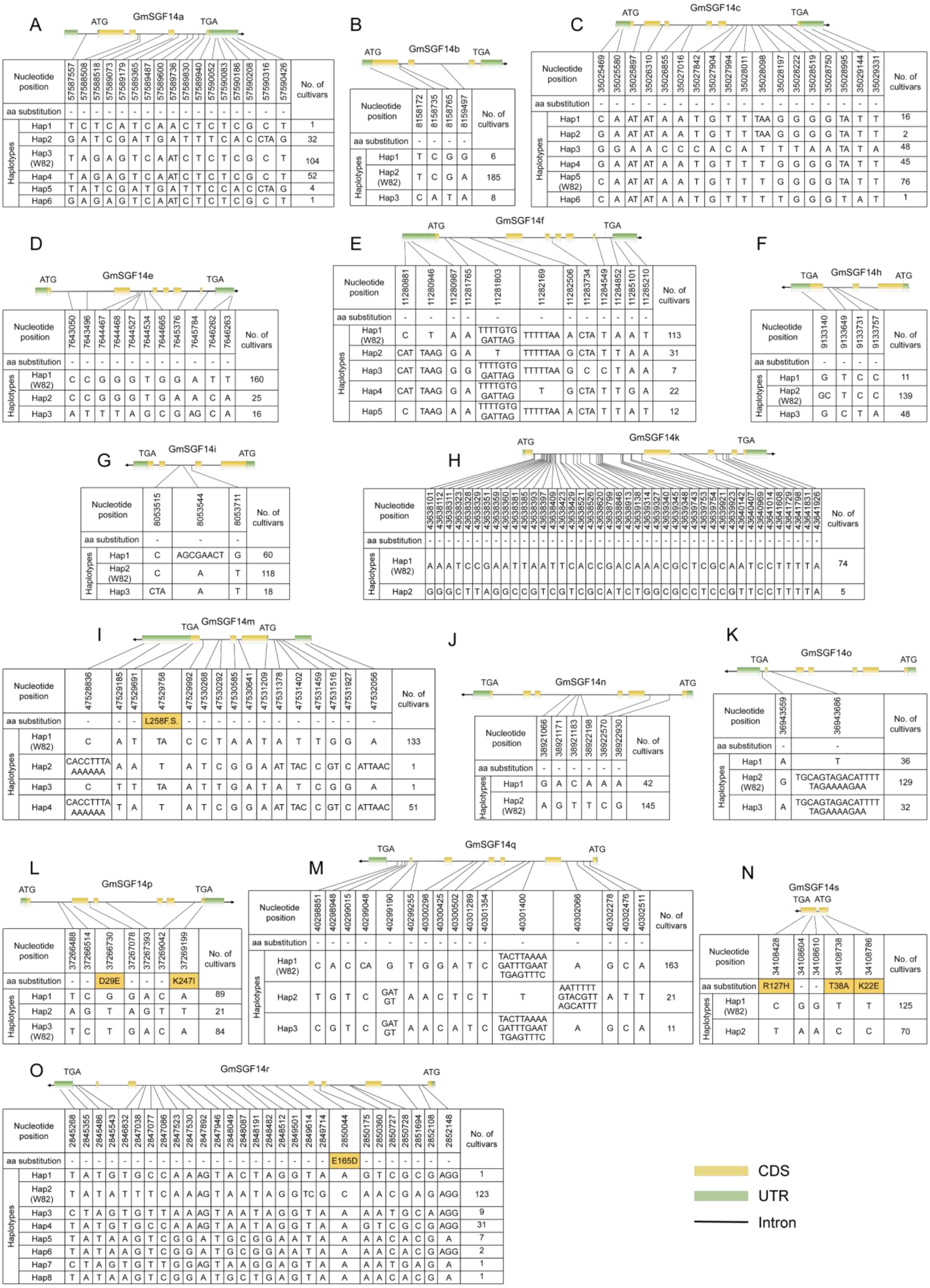

Figure S2. Haplotype analysis of *GmSGF14* genes in 207 soybean varieties. . Amino acid changes (aa) are marked in yellow. F.S.: frameshift.

Table S1. Physicochemical properties of GmSGF14 proteins in soybean

Gene name	Gene ID	Amino acid number / aa	Molecular weight/kD	pI	Instability index	Aliphatic index	GRAVY
GmSGF14a	*Glyma.18G298300*	258	29.1	4.70	48.8	89.3	-0.463
GmSGF14b	*Glyma.04G092600*	251	28.2	4.81	42.0	90.2	-0.352
GmSGF14c	*Glyma.05G158100*	260	29.3	4.80	41.6	82.6	-0.475
GmSGF14d	*Glyma.13G290900*	262	29.5	4.77	39.2	82.0	-0.570
GmSGF14e	*Glyma.01G058000*	260	29.5	4.83	50.8	80.7	-0.580
GmSGF14f	*Glyma.02G115900*	260	29.5	4.83	51.7	81.4	-0.580
GmSGF14g	*Glyma.02G208700*	263	29.4	4.67	46.5	82.4	-0.481
GmSGF14h	*Glyma.04G099900*	289	32.4	4.67	51.3	90.2	-0.311
GmSGF14i	*Glyma.06G101500*	280	31.7	5.46	52.0	92.8	-0.366
GmSGF14j	*Glyma.06G094400*	251	28.4	4.81	41.1	90.5	-0.347
GmSGF14k	*Glyma.14G176900*	315	35.2	4.71	44.8	83.7	-0.340
GmSGF14l	*Glyma.08G115800*	260	29.2	4.90	45.0	78.4	-0.553
GmSGF14m	*Glyma.08G363800*	261	29.4	4.81	45.4	102	-0.159
GmSGF14n	*Glyma.12G229200*	266	30.5	4.85	44.8	79.5	-0.598
GmSGF14o	*Glyma.12G210400*	262	29.5	4.73	40.5	82.3	-0.546
GmSGF14p	*Glyma.13G270600*	264	30.2	4.84	46.2	84.9	-0.545
GmSGF14q	*Glyma.07G226000*	260	29.6	4.79	52.4	83.7	-0.583
GmSGF14r	*Glyma.20G025900*	261	29.6	4.79	53.8	81.5	-0.612
GmSGF14s	*Glyma.17G208100*	160	18.7	5.61	41.3	83.3	-0.116
GmSGF14t	*Glyma.20G043700*	71	7.92	5.70	32.3	74.0	-0.180

Table S2. The information of cis-acting elements within promoter regions of *GmSGF14*

cis-acting element	Element action information
TCT-motif	part of a light responsive element
G-Box	cis-acting regulatory element involved in light responsiveness
Box 4	part of a conserved DNA module involved in light responsiveness
circadian	cis-acting regulatory element involved in circadian control
chs-CMA1a	part of a light responsive element
GATA-motif	part of a light responsive element
GCN4_motif	cis-regulatory element involved in endosperm expression
O2-site	cis-acting regulatory element involved in zein metabolism regulation
AE-box	part of a module for light response
G-box	cis-acting regulatory element involved in light responsiveness
TGA-element	auxin-responsive element
Box II	protein binding site
AuxRR-core	cis-acting regulatory element involved in auxin responsiveness
GC-motif	enhancer-like element involved in anoxic specific inducibility
chs-CMA2a	part of a light responsive element
I-box	part of a light responsive element
TCCC-motif	part of a light responsive element
GT1-motif	light responsive element
AT1-motif	part of a light responsive module
TGA-box	part of an auxin-responsive element
AACA_motif	involved in endosperm-specific negative expression
ATCT-motif	part of a conserved DNA module involved in light responsiveness
3-AF3 binding site	part of a conserved DNA module array (CMA3)
MSA-like	cis-acting element involved in cell cycle regulation
LAMP-element	part of a light responsive element
GA-motif	part of a light responsive element
CAT-box	cis-acting regulatory element related to meristem expression
MRE	MYB binding site involved in light responsiveness
LS7	part of a light responsive element
Sp1	light responsive element
Gap-box	part of a light responsive element
3-AF1 binding site	light responsive element
CAG-motif	part of a light response element
ACE	cis-acting element involved in light responsiveness
AAAC-motif	light responsive element
TATC-box	cis-acting element involved in gibberellin-responsiveness
P-box	gibberellin-responsive element

Table S3. Source information of 207 re-sequenced soybean varieties

Cultivar	Region	Source	Cultivar	Region	Source
Beihudou	NE	Heilongjiang	Heinong44	NE	Heilongjiang
Beifeng2	NE	Heilongjiang	Heinong48	NE	Heilongjiang
Beifeng9	NE	Heilongjiang	Kenfeng16	NE	Heilongjiang
Beifeng11	NE	Heilongjiang	Suinong8	NE	Heilongjiang
Beidou5	NE	Heilongjiang	Suinong10	NE	Heilongjiang
Dengke1	NE	Heilongjiang	Changnong4	NE	Jilin
Dongnong4	NE	Heilongjiang	Miquanhuangdou	NE	Xinjiang
Dongnong72-806	NE	Heilongjiang	Changjihuangdou	NE	Xinjiang
Fengshou10	NE	Heilongjiang	Zhi2	NE	Jilin
Fengshou12	NE	Heilongjiang	Zihua4	NE	Heilongjiang
Fengshou17	NE	Heilongjiang	Changnong5	NE	Jilin
Fengshou19	NE	Heilongjiang	Fengdihuang	NE	Jilin
Fengshou24	NE	Heilongjiang	Huangbaozhu	NE	Jilin
Hefeng25	NE	Heilongjiang	Jilin3	NE	Jilin
Hefeng35	NE	Heilongjiang	Jilin6	NE	Jilin
Heihe3	NE	Heilongjiang	Jilin13	NE	Jilin
Heihe9	NE	Heilongjiang	Jilin20	NE	Jilin
Heihe18	NE	Heilongjiang	Jilin30	NE	Jilin
Heihe19	NE	Heilongjiang	Jilin47	NE	Jilin
Heihe27	NE	Heilongjiang	Jiti5	NE	Jilin
Heihe38	NE	Heilongjiang	Jiyu57	NE	Jilin
Heihe51	NE	Heilongjiang	Jiunong9	NE	Jilin
Heihe54	NE	Heilongjiang	Jiunong22	NE	Jilin
Heilongjiang41	NE	Heilongjiang	Xiaojinhuang1	NE	Jilin
Huajiang4	NE	Heilongjiang	Zaofeng1	NE	Jilin
Jinyuan2	NE	Heilongjiang	Jiti1	NE	Liaoning
JianMGodou1	NE	Heilongjiang	Jin6604-24	NE	Liaoning
Jingshanpu	NE	Heilongjiang	Kaiyu3	NE	Liaoning
Kexi283	NE	Heilongjiang	Kaiyu8	NE	Liaoning
Kangxian4	NE	Heilongjiang	Kaiyu10	NE	Liaoning
Mancangjin	NE	Heilongjiang	Tiefeng3	NE	Liaoning
Suinong3	NE	Heilongjiang	Tiefeng8	NE	Liaoning
Suinong28	NE	Heilongjiang	Tiefeng18	NE	Liaoning
Suinong41	NE	Heilongjiang	Tiefeng19	NE	Liaoning
Mengdou30	NE	Inner Mongolia	Tiefeng20	NE	Liaoning
Hejiao6	NE	Heilongjiang	Fushou	NE	Inner Mongolia
Hejiao8	NE	Heilongjiang	Jindou2	NE	Shanxi
Hefeng22	NE	Heilongjiang	Jindou3	NE	Shanxi
Hefeng45	NE	Heilongjiang	Dandou2	NE	Liaoning
Hefeng47	NE	Heilongjiang	Dandou4	NE	Liaoning
Hefeng50	NE	Heilongjiang	Jin8-14	NE	Liaoning
Hefeng55	NE	Heilongjiang	Jindou33	NE	Liaoning
Heinong16	NE	Heilongjiang	Liaodou15	NE	Liaoning
Heinong26	NE	Heilongjiang	Tiefeng31	NE	Liaoning
Heinong33	NE	Heilongjiang	Dandou1	NE	Liaoning
Heinong35	NE	Heilongjiang	Tiefeng29	NE	Liaoning
Heinong37	NE	Heilongjiang	Changpingqingdou	NE	Beijing
Heinong43	NE	Heilongjiang	Clay	USA	USA

Cultivar	Region	Source	Cultivar	Region	Source
McCall	USA	USA	Hezeniumaohuang	HHH	Shandong
Merit	USA	USA	Ludou4	HHH	Shandong
Norman	USA	USA	Yanhuang1	HHH	Shandong
Portage	USA	USA	Yidupingdinghuang	HHH	Shandong
Wilkin	USA	USA	Wenfeng5	HHH	Shandong
Chippewa64	USA	USA	Wenfeng7	HHH	Shandong
Evans	USA	USA	Shangcaiercaoshipingdingshi	HHH	Henan
Amsoy	USA	USA	Weiqingdou	HHH	Henan
Amsoy71	USA	USA	Zhengzhou135	HHH	Henan
Beeson	USA	USA	Naiyinheidou	HHH	Hebei
Corsoy	USA	USA	Haiyangpamanqing	HHH	Shandong
Hark	USA	USA	Yuejin4	HHH	Shandong
Harosoy63	USA	USA	Yudou2	HHH	Henan
Wayne	USA	USA	Yudou8	HHH	Henan
Clark	USA	USA	Yudou22	HHH	Henan
Cutler	USA	USA	Zheng92116	HHH	Henan
Bedford	USA	USA	Xudou1	HHH	Jiangsu
Dare	USA	USA	Xudou2	HHH	Jiangsu
Forrest	USA	USA	Xudou5	HHH	Jiangsu
Centennial	USA	USA	Xudou9	HHH	Jiangsu
Hood	USA	USA	58-161	HHH	Jiangsu
Tracy	USA	USA	Wandou24	HHH	Aahui
Bragg	USA	USA	Yuejin5	HHH	Shandong
Braxton	USA	USA	Taixingheidou	SC	Jiangsu
Dowling	USA	USA	Edou8	SC	Hubei
Jupiter	USA	USA	Aijiaozao	SC	Hubei
Huairouhuangdou	HHH	Beijing	Tianlong1	SC	Hubei
Qunyingdou	HHH	Hebei	Guichun1	SC	Guangxi
Zhonghuang30	HHH	Hebei	Heibiqing	SC	Guangdong
Zhonghuang35	HHH	Hebei	Zigongqingpidou	SC	Sichuan
Jindou19	HHH	Shanxi	Liuyuehuang	SC	Guizhou
Jindou21	HHH	Shanxi	Qiandou6	SC	Guizhou
Jindou25	HHH	Shanxi	Jinjiangdaqingren	SC	Fujian
Bahong1	HHH	Hebei	Taiwan75	SC	Taiwan
Youbian30	HHH	Hebei	Guichun8	SC	Guangxi
Jindou23	HHH	Shanxi	Baihuadou	SC	Guangdong
Hai94	HHH	Shanxi	Yulingdahuangdou	SC	Guangxi
Jidou7	HHH	Hebei	Juhuangdadou	SC	Guangdong
Ludou11	HHH	Shandong	Wuhuasiyuehuang	SC	Guangdong
Qihuang10	HHH	Shandong	Shangyukanshanbai	SC	Zhejiang
Henanzaofeng1	HHH	Henan	Zhechun3	SC	Zhejiang
Kefeng6	HHH	Hebei	Chengliuniumaohuang	SC	Henan
Handou5	HHH	Hebei	Fengchengniupidou	SC	Jiangxi
Jidou12	HHH	Hebei	Yunyizao	SC	Hunan
Jidou17	HHH	Hebei	Dian86-4	SC	Yunnan
Zhongdou19	HHH	Hebei	Jinningdahuangdou	SC	Yunnan
Zhonghuang13	HHH	Hebei	Suidaohuang	SC	Jiangsu
Zhonghuang37	HHH	Hebei	Edou2	SC	Hubei
Hedou13	HHH	Shandong	Houzimao	SC	Hubei

Cultivar	Region	Source	Cultivar	Region	Source
Nannong493-1	SC	Jiangsu	Yangchunqingdou	SC	Guangdong
Suxie1	SC	Jiangsu	Lanxidaqingdou	SC	Zhejiang
Bayueqing	SC	Hunan	Zigongdongdou	SC	Sichuan
Jingda332	SC	Jiangsu	Shangraodaqingsi	SC	Jiangxi
Pingguohuangdou	SC	Guangxi	Qiudou1	SC	Hunan
Nandou12	SC	Sichuan			

* NE: Northeast of China; HHH: Huang-Huai-Hai; SC: South of China.

Table S4. Primer sequences used for qRT-PCR analysis of *GmSGF14* gene family

Gene name	Forward primer (5′-3′)	Reverse primer (5′-3′)
GmSGF14a	CCGCATCTCCCGAGAGTAAA	CCAGCCTAATGGGGTGAGTG
GmSGF14b	CGAGTTTAAGGTTGGGGACGAA	GTTGAGAGCAAGACCCAATCT
GmSGF14c	AAAGGGAACGATGTGAGCGT	TCACCGGACTTGAACTCTGC
GmSGF14d	CGTCTACATCGCCAAACTCG	ATCCTCCAGGACGCTCTGC
GmSGF14e	GGCGCATCATGTCCTCAATT	GCTGATGCGGAAGAAGGAAT
GmSGF14f	CCTCTTGGCGCATAATGTCC	TCTGCTGATCCGGAAGAAGG
GmSGF14g	CTATCAGGCTGGGTCTTGCT	CATCTGCACCATCATCCTGC
GmSGF14h	AGGTTGCCATAGCTCATCCA	ATCCTGTTTCGGTGCTGCTT
GmSGF14i	AACATCTGCGACGGTATCCT	ATGAGTAGGAGGCAGTTCGG
GmSGF14j	TCAACCTCCTCGACTCCAAC	AAGTTGAGAGCGAGACCCAA
GmSGF14k	GCGACGGAATCCTCAAACTC	ACACGGAGAAGTTCAGAGCA
GmSGF14l	GCTGAACGTGGAATTGACGG	ACCAGCAGAGCTAGAGGGAA
GmSGF14m	CTCATTCCTTCCGCTGCATC	TTGAGAGCAAGTCCCAACCT
GmSGF14n	CGGAGAGCTTCATGGAGGAT	CCAGCCTTGAATTCTGCCAA
GmSGF14o	CTTCGTCTACATCGCCAAGC	CTGCCTGTACTCCTTAATCCG
GmSGF14p	TCGGAGCTGGATACCCTGAA	TCTCCTTGATCAACCCTGGC
GmSGF14q	CTTGGCGCATCATGTCATCA	TCCTGGTCGGTCTTGAACTC
GmSGF14r	GGACTTGCGCTCAACTTCTC	GCAGGTTTGGCTTCTTCTGT
GmSGF14s	CCGTCGCCTACAAGAACATG	GGCTCCCTTCATCTTGAGGT
GmSGF14t	TGTTGAAATGTTCTGCTTGCA	AGGTAAATCAACCTCTGCTGCAGT
GmActin	CGGTGGTTCTATCTTGGCATC	GTCTTTCGCTTCAATAACCCTA

* *GmActin*: *Glyma18g52780*, was used as the internal reference.
